# Decisions of Value: Going Backstage: Comment on "Contextual Factors Influencing Cost and Quality Decisions in Health and Care: A Structured Evidence Review and Narrative Synthesis"

**DOI:** 10.15171/ijhpm.2018.81

**Published:** 2018-08-25

**Authors:** Michael Calnan

**Affiliations:** Social Policy, Sociology and Social Research (SSPSSR), University of Kent, Canterbury, UK.

**Keywords:** Priority Setting, Decision-Making, Uncertainty, Pragmatis, English NHS

## Abstract

This commentary expands on two of the key themes briefly raised in the paper involving analysis of the evidence about key contextual influences on decisions of value. The first theme focuses on the need to explore in more detail what is called backstage decision-making looking at how actual decisions are made drawing on evidence from ethnographies about decision-making. These studies point to less of an emphasis on instrumental and calculative forms of decision-making with more of an emphasis on more pragmatic rationality. The second related theme picks up on the issue of sources of information as a contextual influence particularly highlighting the salience of uncertainty or information deficits. It is argued that there are a range of different types of uncertainties, not only associated with information deficits, which are found particularly in allocative types of decisions of value. This means that the decision-making process although attempting to be linear and rational, tends to be characterised by a form of navigation where the decision-makers navigate their way through the uncertainties inherent and overtly manifested in the decision-making process.

The importance of understanding the contextual influences on decision-making about cost and quality related questions in the organisation and provision of healthcare is well recognised.^1^ However, this paper^2^ goes one stage further by carrying out a structured evidence review and narrative synthesis trying to identify the evidence from the international available literature about the key contextual influences. A distinction is made between allocative and technical types of decisions of value with the bulk of evidence being found in relation to the former rather than the latter type of decision-making. The analysis, drawing on the framework provided by Pettigrew,^3^ identifies a number of inner and outer contextual influences on what the authors call ‘decisions of value.’ In terms of the contextual influences these are categorised in terms of sources of information; interests; organisational characteristics, governance and leadership, geography, economics and relationship to government. The focus is at the meso level, as opposed to the micro- and macro-levels, and on more formal aspects of decision-making.

The paper provides a useful presentation of the state of the evidence about contextual influences but tends to pay limited attention to what might be called backstage^4^ as opposed to front stage decision-making. This is alluded to in the paper but it is central to understanding how decisions get made. For example, in the area of priority setting these decision-making processes have been described, at least some time ago, as ‘muddling through elegantly’ where there is more evidence of negotiation rather than rationality or instrumentality in decision-making.^5^ This type of decision-making process is messy and non-linear, and in spite of apparent significant changes in the quality of evidence available and the sophistication of techniques used to analyse these data, has still been found at different levels of decision-making in the public funded national health service in England. For example, research focusing at the national level involving the ‘fourth’ stage of medicine regulation and which has explored decision-making by the National Institute for Health and Care Excellence (NICE) about the appraisal of expensive medicines has identified the difference between front stage and backstage decision-making. The discourse associated with front stage decision-making emphasises the dominant influence of the technical criteria of cost-effectiveness although in some cases social values tended to receive some explicit recognition in the decision-making such as in the treatment for younger children. The attempt to explicitly incorporate social and ethical values was shaped by an approach described as ‘accountability for reasonableness’ which emphasised the conditions of transparency, relevance, and revisabilty.^6^ Evidence about the implementation of policies in some countries based on this ‘accountability for reasonableness’ approach is available.^7^ However, this evidence tends to focus on decision-making at the formal level and the research evidence from ethnographic studies involving interviews, documentary analysis and observation points suggests that while the discourse particularly on cost-effectiveness did generally frame the approach taken a less than rational or calculative approach in the backstage decision-making was prevalent. This research identifies the implicit social influences about how decisions are made and suggest that the decision-making process is characterised by a form of navigation, (rather than ‘muddling through’) where the decision-makers navigate their way through the uncertainties inherent in what is formally described as evidence-based decision-making process.^8^ The paper suggests that *‘cost effectiveness analysis which has been applied with some success to allocative decision-making at a macro level’* (p. 11).^2^ The evidence from ethnographic studies^9^ suggest that this account may only present a partial picture of the nature of the decision-making process and what shapes it.

Similarly at the more local level decisions about the commissioning might also be characterised as practical rationality and involve intuition and experiential knowledge^9^ and a ‘case and judgement based’ approach.^10^ In both these national and local level contexts the use of practical rationality is evident but appeared to complement the dominant instrumental discourse, although in the local context emphasis in the discussion on ethical issues in relation to the allocation of resources was not only more overt but related more directly to individual circumstances.

A related issue is the question of sources of information which is identified as one of the key contextual elements. The paper identifies the importance of the absence of information ‘*high levels of uncertainty in the face of information deficits have been shown to reduce adherence to an instrumental decision-making model and to open up determinations to greater levels of judgement and intuition’* (p. 9).^2^ Thus, there is recognition of the salience of uncertainty in the context of these decision-making and the implications for rational decision-making. However, studies have shown that in allocative decision-making there are different types of uncertainties not only associated with information deficits and these will need to be recognised and be managed if a decision is to be made. Three different types of uncertainty have been identified which are interrelated in the decision-making process^11^ which were epistemic (referring to the ability of biomedical methods used by the pharmaceutical industry to produce knowledge about treatments), procedural (particularly relating to the sheer volume of evidence considered), and interpersonal (which refers to the competency and motives of those providing evidence such as the representatives from the pharmaceutical industry and clinical experts). There was also uncertainty and ambiguity associated with the level of technicality and complexity of the information provided.^8^ Agencies such as NICE recognise, attempt to address and try to resolve some of these epistemological uncertainties particularly through quantitative techniques.^12^ However, the evidence^8^ also suggested that navigation of these layers of uncertainty was (partially) managed through practical rationality and various forms of trust at different levels. Trust was one of a number of means used to bridge uncertainty. Both individual decision as rules of thumb and collective strategies were evident in the management of uncertainty in the decision-making process. Thus, though seemingly an objective techno-scientific evaluation, social forces necessarily emerge in the development and subsequent management of uncertainty.^8^

There is some disagreement over how these uncertainties should be tackled although there is consensus that they should be recognised and acknowledged rather than ignored and being bracketed off. However, while one approach tends to want to minimise them as they are seen as a problem^13^ whereas the other see uncertainty more positively as a way of making rationing decisions more transparent, accountable and democratic.^14^

The review paper^2^ identifies the significance of external and internal interests but says little about the key role of commercial interests in influencing decision-making even though some appear to have been identified in the papers reviewed. It might be argued that the profit motive which might be the primary driver of these commercial interest groups which could be at odds with the public interest and the professional values of those providing the healthcare. This would include the influence of corporate private companies who finance and provide healthcare and of the multi-national pharmaceutical industry. For example, the study^8^ previously described also illustrated the potential risks of regulatory capture^15,16^ of NICE in England by the pharmaceutical industry although there are both formal and informal mechanisms to attempt to manage and resist their influence. In this case the pharmaceutical industry might be characterised as both an external and internal contextual influence given that it contributes to the process by providing and controlling access to evidence about cost effectiveness but is not directly involved in the decision-making.

More generally, the organising framework developed by Pettigrew^3^ based on an organisation outside of health system in the industrial sector is used to analyse the difference between external and internal contextual influences. It must be emphasised that this framework relates primarily to health systems in high income countries and tends to focus on, although not explicitly stated, organisational and political influences rather than cultural context.^17,18^ This is a useful descriptive schema for categorising and classification but as the authors suggest it is a framework mainly used for analysing change processes rather than explaining the relative importance of different layers of contextual influence. Certainly, it is difficult to assess the explanatory power of the framework given that it was not specifically designed for this particular purpose. One area that needs to be discussed in more depth is the dynamic nature of the decision-making process and the interrelationship between the different layers of the influence. These may be at the macro, meso and micro levels and the question is which are the most powerful contextual influences? Alternative theoretical approaches such as the structural interest approach of Alford^19^ might shed more light on this. The paperproposes^2^ that the evidence suggests that internal influences appear to be more powerful although much depends upon the latitude available to local actors in their decision-making. Local managers and clinicians, at least in the National Health Service (NHS) in England, tend to have some degree of relative autonomy and discretion but it has been suggested that the interplay between corporate monopolisers and professional rationalisers^19,20^ might shape the decision-making process in many healthcare organisational settings which in turn could limit in particular the influence of bottom-up pressures.

The influence of bottom up pressures is raised in the paper^2^ through discussion of the role of the patients in decision-making and the importance of hearing the patient voice. This should certainly help democratise health services and mitigate against the dominance of managerial and professional interests as well enhance patient centred care and the coproduction of knowledge.^14^ However, it has proved difficult sometimes to square specific patients interests with more general decisions about the allocation of resources and disinvestment decisions ie, what benefits the specific patient group may not be beneficial for the population as a whole.^21,22^

Finally, from a methodological point of view the studies discussed here have tended to adopt ethnographic designs although those reviewed in the paper seem to be short on the use of this type of methodology involving observation to directly understand how and why decisions are made in everyday contexts. The lack of such studies creates considerable limitations for gaining insights into understanding the nature of decision-making and its evidence base.

## Ethical issues

Not applicable.

## Competing interests

Author declares that he has no competing interests.

## Author’s contribution

MC is the single author of the paper.
